# Magnetic Resonance Imaging of Inner Ear and Internal Auditory Canal Structures in the Presence of a Cochlear Implant

**DOI:** 10.1097/MAO.0000000000004445

**Published:** 2025-02-19

**Authors:** Rebecca Susan Dewey, Robert A. Dineen, Matthew Clemence, Nitin Menon, Richard Bowtell, Patrick Boyle, Douglas E. H. Hartley

**Affiliations:** ∗ Sir Peter Mansfield Imaging Centre, School of Physics and Astronomy, University of Nottingham, NG7 2RD, UK; † Hearing Sciences, Mental Health and Clinical Neurosciences, School of Medicine, University of Nottingham, NG7 2UH, UK; ‡ National Institute for Health Research, Nottingham Biomedical Research Centre, Nottingham, NG1 5DU, UK; § Radiological Sciences, Mental Health and Clinical Neurosciences, School of Medicine, University of Nottingham, NG7 2UH, UK; ∥ Philips Healthcare, Best, Netherlands; ¶ Nottingham University Hospitals NHS Trust, Queens Medical Centre, Nottingham, NG7 2UH, UK; ∗∗ Advanced Bionics GmbH, Hanover D-30625, Germany; †† Rinri Therapeutics Ltd., Innovation Centre, 217 Portobello, Sheffield S1 4DP, UK

**Keywords:** Cochlear implant, Image artifact, Magnetic resonance imaging, Postoperative surveillance

## Abstract

**Objective:**

To determine whether the internal auditory canal (IAC) can be visualized using magnetic resonance imaging (MRI) in users of a cochlear implant (CI) model that can safely undergo MRI at 3 T.

**Patients:**

Four normally hearing controls and three individuals unilaterally implanted with a HiRes Ultra 3D (Advanced Bionics LLC, California, USA).

**Interventions:**

Participants underwent 3 T MRI using sequences appropriate for the postoperative surveillance of the IAC. Images in normally hearing individuals were acquired after placing a fully functional, unpowered, CI underneath a swimming cap at each of eight candidate scalp positions, four on each side of the head. Images were compared to a control condition without a CI present. and CI users were imaged with similar sequences.

**Main Outcome Measures:**

In normally hearing controls, the likely impact of the artifact on detection of pathology for multiple neuroradiological locations as rated by two independent radiologists. In CI users, a qualitative assessment of the diagnostic usability of images.

**Results:**

Visibility of the ipsilateral IAC and cochlea varied among the three CI users, with images from one participant deemed largely usable, while those from the other two participants exhibited less diagnostic certainty, likely due to differences in implant locations and cranial/neuroanatomical variations. Ratings of images in normally hearing participants showed that more middle-to-anterior CI locations were associated with reduced likelihood of overlooking gross abnormalities.

**Conclusion:**

Through meticulous surgical placement, bilateral IAC visualization may be achievable for monitoring chronic health conditions such as tumor surveillance in high-risk patients, and as a safety monitoring outcome measure in clinical trials.

## INTRODUCTION

Magnetic resonance imaging (MRI) facilitates the identification of suspected abnormalities of the membranous inner ear structures and internal auditory canals (IAC). It provides excellent contrast between soft tissue and cerebrospinal fluid (CSF)/endolymph/perilymph, combined with high spatial resolution, as well as contrast enhancement of relevant pathologies. Furthermore, MRI provides the capacity to employ multiple different contrast mechanisms in a single session.

A cochlear implant (CI) comprises a retaining magnet that aligns the radiofrequency coils of internal and external device components through the scalp. In recent years, several CI manufacturers have introduced implant models with retaining magnets that rotate under the influence of an external magnetic field, such as that of an MR scanner. This significantly reduces the torque experienced by the retaining magnet, thereby negating the need to remove the magnet for MRI and improving the safety and comfort of the CI user undergoing MRI. Consequently, it is now both plausible and ethical to include CI users as participants in MR research studies, enabling MR imaging and/or spectroscopy to address research questions with no immediate or direct benefit to the participant. Nevertheless, imaging the head is still challenging in this group, due to the substantial image artifacts caused by the presence of the retaining magnet (1,2). Additionally, CI positioning (3,4) affects which anatomical features are obscured.

Indications for MRI of the IACs and inner ear structures in CI users include known or suspected tumors in the cerebellopontine cistern, such as in neurofibromatosis type 2 (5–8), and assessing novel therapies for hearing loss. Recent advancements in cell therapy suggest the potential of otic neural progenitors to regenerate damaged auditory nerves in individuals with profound hearing loss (9). The next developmental step in making this therapeutic intervention widely available is conducting a first-in-human study to assess the safety and tolerability of this cell therapy, which would be offered in addition to a CI in a group of patients with neural deafness. Given the theoretical risk of tumorigenesis associated with neural progenitors, identifying an appropriate imaging protocol for postoperative safety surveillance is imperative (10). Such surveillance, required at multiple timepoints following surgery, renders removal of the retaining magnet for imaging far from ideal.

Several studies have assessed image artifacts associated with MR-conditional CIs. For example, Majdani et al. (11) observed image artifacts using 1.5 and 3 T MRI in cadavers and at 1.5 T in patients using MedEl Synchrony and Advanced Bionics HiRes 90 K devices. They found that the image artifact associated with this device covered most of the cranial hemisphere ipsilateral to the cochlear implant. Subsequently, Canzi et al. (12) evaluated the MRI-induced artifacts produced by an Advanced Bionics Ultra 3D CI in three human cadaveric heads. Three blinded observers found that images of the ipsilateral cochlea, IAC, and auditory brainstem acquired at 1.5 T were all of high quality. More recently, Dewey et al. (4) studied image artifacts using 3 T MRI in normally hearing individuals with a Cochlear CI612 CI placed under a swimming cap in different locations. They found that the image artifact only partially obscured the side ipsilateral to the CI, particularly when the magnet was placed in a posterior position. However, this study did not specifically address the visibility of the ipsilateral cochlea, IAC, or auditory brainstem, and the sequence assessed was not one that is used routinely in diagnostic imaging of the IAC and inner ear. Although image artifacts induced by the presence of the implant are expected to be larger at 3 T than at 1.5 T, the higher field strength promises superior tissue contrast and spatial resolution, ensuring that the resulting images are sufficient to provide confidence in postoperative surveillance in these individuals.

This report aims to investigate imaging artifacts from a CI with a rotating design retaining magnet, and to provide a clinically relevant optimized MRI acquisition protocol for detecting IAC tumors in a future trial of otic neural progenitor therapy combined with a CI. The current study seeks to assess the impact of the implant location on the usability of optimized clinical sequences for postimplantation surveillance of bilateral IACs, and to provide outcome measures for human clinical trials of novel adjunctive therapies, including cell-based treatments (9).

## MATERIALS AND METHODS

The study methods are described in the Supplementary Materials, http://links.lww.com/MAO/C47, including Supplementary Figure 2A, http://links.lww.com/MAO/C50 and Supplementary Table 1, http://links.lww.com/MAO/C48.

## RESULTS

Results of the phantom study (Supplementary Fig. 1, http://links.lww.com/MAO/C49) and the quantitative (Supplementary Fig. 2B, http://links.lww.com/MAO/C50) and qualitative (Supplementary Fig. 3, http://links.lww.com/MAO/C51, with inter-rater comparison in Supplementary Table 2, http://links.lww.com/MAO/C48) assessment of artifact size and radiological evaluation of diagnostic impact of artifacts for different CI locations in normally hearing participants are provided in the Supplementary Materials, http://links.lww.com/MAO/C47.

### Safety and Tolerability

All three CI users tolerated the entire scanning procedure, with no participant reporting any effects or sensations relating to undergoing MRI, or any reduced CI function or retaining magnet strength following scanning.

### Imaging in CI Users: T2DRIVE

**FIG. 1:**
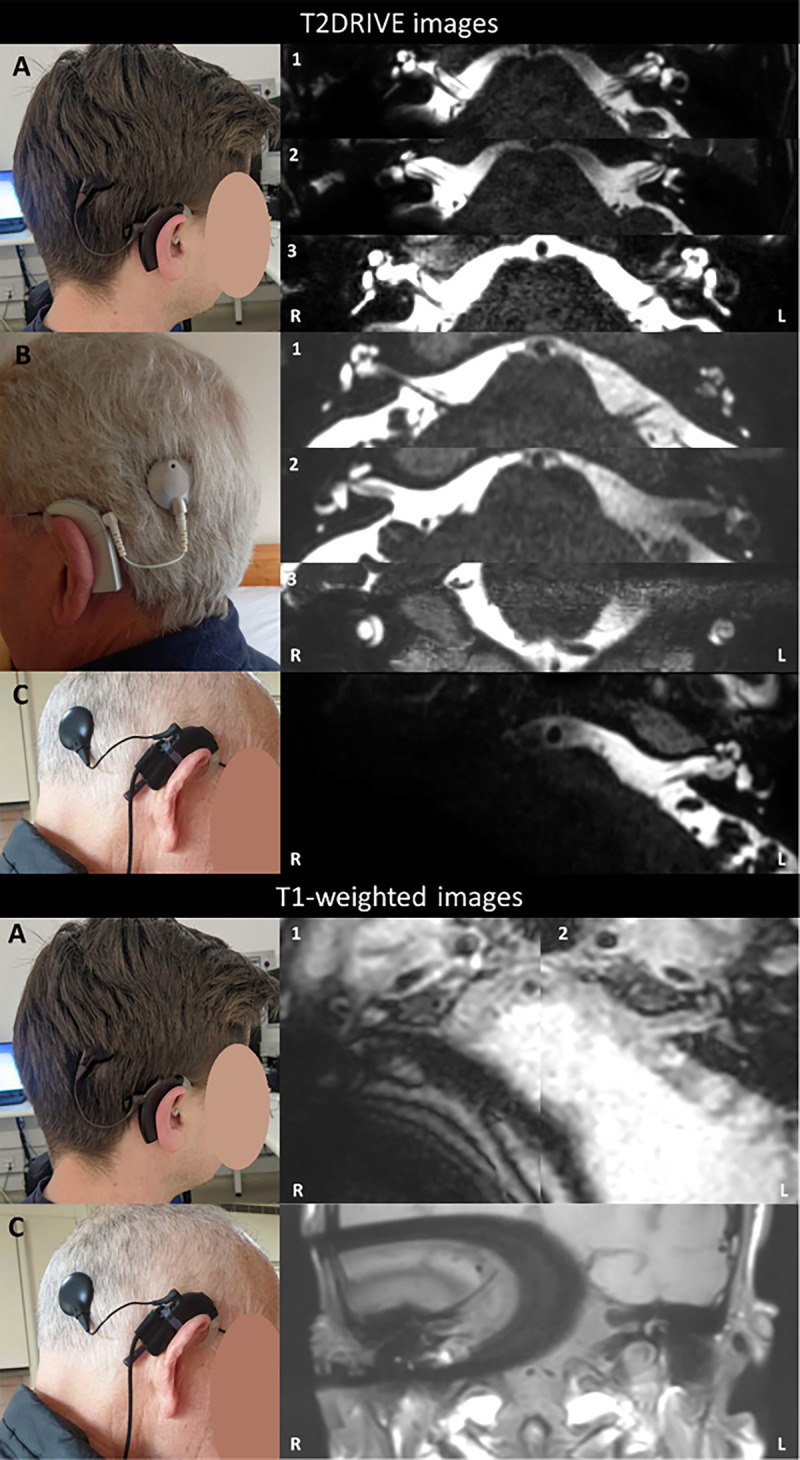
*Top*, T2DRIVE images in three CI users (A, B, and C). In participant A, transverse slices through the bilateral IACs are shown without MARS (1 and 2) and with MARS (3). In participant B, transverse (1 and 2) and coronal (3) are shown. In participant C, a transverse slice is shown. *Bottom*, T1-weighted images are shown in two CI users (A and C). In participant A, transverse slices through the bilateral IAC are acquired using a T1-weighted TSE with mDIXON and no metal artifact reduction. In participant C, a transverse slice is shown, acquired using a T1-weighted TSE with SPIR and SEMAC.

Figure 1 (*top*) shows T2DRIVE images acquired in three CI users. A full description of these images is given in the Supplementary Materials, http://links.lww.com/MAO/C47.

### Imaging in CI Users: T1-Weighted TSE with Fat Suppression

Figure 1 (*bottom*) shows T1-weighted TSE with fat suppression images acquired in the two CI users that imaging was at least partly usable in. A full description of these images is given in the Supplementary Materials, http://links.lww.com/MAO/C47.

## DISCUSSION

Benefits of MRI in CI users include monitoring for tumors in individuals with neurofibromatosis type 2 and tracking the effects of new therapies for hearing loss. For the latter, this work was motivated by the need to develop a protocol for the surveillance of patients enrolled in a future clinical trial of an otic neural progenitor cell therapy as an adjunct to cochlear implantation. Magnet removal and reinsertion to reduce the MRI artifact associated with a CI risks infection and has a cumulative detrimental impact on the scalp covering the implant. Therefore, it is impractical to perform magnet removal for repeated MR scanning, such as would be required for serial tumor surveillance over time. Selecting an optimal surgical insertion site for the CI, to minimize MRI artifacts, based on individual preoperative evaluation of artifact location as described in this study and previously (4), would be highly beneficial for facilitating postimplantation surveillance without the need for regular surgical intervention. This presurgical assessment can be performed within an hour with no adverse effects.

The current study was focused on the visualization of the ipsilateral IAC and cochlea, as these are the points most vulnerable to postsurgical tumor generation. Visibility of these structures varied across three CI users, with images in participant A being broadly usable, participant B being somewhat usable, and participant C being more challenging and providing less confidence. This variation is likely due to differences in implant locations and cranial/neuroanatomical variations. Ratings of images in normally hearing participants showed that middle-to-anterior CI locations were associated with lower likelihoods of missing a gross abnormality. Individual presurgical assessment using these methods may be beneficial to maximize the suitability of these selected MRI sequences for assessing bilateral IACs in the monitoring of chronic health conditions, such as tumor surveillance in high-risk patients, and for safety monitoring in clinical trials.

The current study used two different image contrast mechanisms, identified through discussion with an experienced neuroradiologist, improving on previous work (4) that used only one image type (a 3D-T1-weighted gradient echo sequence acquired at 1.5 mm^3^). This allows us to further demonstrate the proof-of-principle for using a swimming cap to assess the impact of multiple implant locations prior to surgery. We have shown that two clinically diagnostic sequences using TSE readouts are only moderately affected by the presence of the CI retaining magnet. However, echo planar-based sequences such as diffusion-weighted imaging and gradient echo-based susceptibility-weighted imaging will be more significantly detrimentally impacted by the presence of metallic implants. To generalize these findings to a broader set of clinical and research questions, it will be necessary also to understand the distribution of CI-induced artifacts for these sequences to inform device placement for CI candidates who are likely to need follow-up MRI reliant on such sequences.

While most CI users will be scanned at the lower field strength of 1.5 T, newer implants with rotating retaining magnets enable conducting both clinical and research MR at 3 T safely, ethically, and comfortably. This study has demonstrated the feasibility and utility of conducting MRI both preoperatively to inform surgical planning in patients where routine MRI acquisition is anticipated or indicated, and postoperatively for postsurgical surveillance. As the procedures described in this article were well tolerated and presented no adverse effects in healthy volunteers, including CI users, there is now no need to avoid conducting further research studies at 3 T in CI users with rotating magnets.

## CONCLUSION

Visibility of the ipsilateral IAC and cochlea varied across three CI users, with images from one participant being broadly usable while in the other two provided less confidence in their diagnostic imaging capacity, likely due to differences in implant locations and cranial/neuroanatomical variations. Ratings of images from normally hearing participants indicated that middle-to-anterior CI locations were associated with a lower likelihood of missing a gross abnormality. Individual presurgical assessments using these methods may maximize the suitability of MRI for evaluating bilateral IACs in monitoring of chronic health conditions, such as tumor surveillance in high-risk patients, and for safety monitoring in clinical trials.
